# Binding of herpesvirus entry mediator (HVEM) and HSV-1 gD affect reactivation but not latency levels

**DOI:** 10.1371/journal.ppat.1011693

**Published:** 2023-09-22

**Authors:** Ujjaldeep Jaggi, Shaohui Wang, Kevin R. Mott, Homayon Ghiasi

**Affiliations:** Center for Neurobiology and Vaccine Development, Department of Surgery, CSMC - SSB3, Los Angeles, California, United States of America; University of Illinois at Chicago, UNITED STATES

## Abstract

Previously we reported that the HSV-1 latency associated transcript (LAT) specifically upregulates the cellular herpesvirus entry mediator (HVEM) but no other known HSV-1 receptors. HSV-1 glycoprotein D (gD) binds to HVEM but the effect of this interaction on latency-reactivation is not known. We found that the levels of latent viral genomes were not affected by the absence of gD binding to HVEM. However, reactivation of latent virus in trigeminal ganglia explant cultures was blocked in the absence of gD binding to HVEM. Neither differential HSV-1 replication and spread in the eye nor levels of latency influenced reactivation. Despite similar levels of latency, reactivation in the absence of gD binding to HVEM correlated with reduced T cell exhaustion. Our results indicate that HVEM-gD signaling plays a significant role in HSV-1 reactivation but not in ocular virus replication or levels of latency. The results presented here identify gD binding to HVEM as an important target that influences reactivation and survival of ganglion resident T cells but not levels of latency. This concept may also apply to other herpesviruses that engages HVEM.

## Author summary

We report that HSV-1 reactivation from latency is significantly impaired in viruses deficient in gD binding to the mouse HVEM gene. These results indicate that binding of gD to HVEM regulates viral reactivation but not levels of latency. These results identify a gD-HVEM relationship as a novel mechanism that could modulate the equilibrium pathway involved in HSV-1 reactivation.

## Introduction

A hallmark of HSV-1 infection is its ability to establish latency in the neurons of an infected host [1–3]. Once acquired, latent infection demonstrates a lifelong pattern of episodic recurrences [4–6], in which the virus reactivates, travels back to the original site of infection, and causes recurrent disease [7–9]. Due to the pre-existing immune response, herpes stromal keratitis is much more likely to occur following recurrent HSV infection [10,11]. In the U.S., it is estimated that 70–90% of the adult population has antibodies to HSV-1 and/or HSV-2, with approximately 25% showing clinical symptoms [12,13]. Approximately 500,000 people per year suffer recurrent ocular HSV episodes requiring doctor visits, medication, and in severe cases, corneal transplants. Currently no vaccine is available that can either prevent ocular infection or has a safe and effective approach to eliminate latent infections that has been developed.

We previously showed that HSV-1 latency and reactivation are significantly reduced in HVEM^-/-^ mice, suggesting that HVEM is involved in latency-reactivation in ocularly infected mice [14]. HVEM (TNFRSF14) is one of the several routes of entry used by HSV-1 to initiate infection in the host [15]. The virion envelope glycoprotein D (gD) of HSV-1 is the primary viral protein that engages the HVEM molecule [15–17]. In addition to gD, HVEM also binds to LIGHT (TNFSF14), lymphotoxin-α (LTα), CD160, and B and T lymphocyte attenuator (BTLA) [18–21]. In contrast to HVEM^-/-^ mice [14], the levels of reactivation in BTLA^-/-^, LIGHT^-/-^, CD160^-/-^, and LTα^-/-^ mice infected with LAT(+) and LAT(-) viruses were similar to WT mice but faster than in HVEM^-/-^ mice infected similarly [22,23].

*In vivo* studies of the mechanisms underlying reactivation are of paramount importance given the complex inter-relationships between replication, latency, and reactivation, as well as the contribution of the microenvironment and immune responses to the status of infected sensory neurons. *In vivo* analyses could be performed using mutated or recombinant viruses. However, the pathogenesis of several HSV-1 strains that are routinely used in experimentation, differ considerably in pathogenesis after ocular infection [24]. Strain McKrae (originally derived from a patient with herpes simplex keratitis) [25] is routinely used by us and many other investigators [26–28]. McKrae is more virulent and undergoes a higher frequency of spontaneous and induced reactivation than strain KOS (originally derived from a patient with a cold sore) [27–31]. Mutant viruses, including KOS-Rid1, KOS-Rid2, and HSV-1 strain ANG have been used to study gD binding to HVEM [16,32,33]. However, in contrast to McKrae, these viruses need corneal scarification due to their lower infectivity *in vivo*.

HSV-1 uses diverse routes of entry to infect different cell types and previously we reported that in LAT(+) infected mice, HVEM mRNA was increased over uninfected mice and while in LAT(-) infected mice, HVEM mRNA was decreased, while there were no significant differences in the mRNA levels of nectin-1, nectin-2, 3-O-sulfated heparan sulfate (3-OS-HS), PILRα or NMHC-IIA in LAT(+) vs LAT(-) infected mice [14]. Both HVEM and nectin-1 are receptors for gD and gD is an essential HSV-1 glycoprotein and in our model of HSV-1 infection, HVEM but not nectin-1 play an important role in latency-reactivation [14]. However, little is known about the gD-HVEM interaction in the context of viral latency and reactivation. Here, we report that HSV-1 reactivation from latency, but not levels of primary infection or latency, is significantly impaired in viruses that lack gD binding to HVEM. Thus, our results indicate that the gD-HVEM interaction regulates reactivation but not viral latency. These results confirm the importance of the gD-HVEM relationship as a major mechanism that may modulate homeostatic pathways involved in HSV-1 latency-reactivation.

## Results

### gD binding to HVEM affects reactivation in mice infected with KOS-Rid1 virus

Until recently, it was generally believed that the HSV-1 genome is maintained in a quiescent state in sensory neurons during latency, i.e. there is an absence of detectable viral protein synthesis. However, in mouse studies, lytic transcripts and proteins may be expressed at very low levels in latently-infected ganglia. For example, expression of HSV-1 proteins, ICP4, tk, gB, and gC have been detected in trigeminal ganglia (TG) of latently-infected mice [34–39]. Human observational data based on tissue sampling further supports the expression of HSV-1 ICP0 and ICP4 transcripts during latency [40,41]. Our published data extend these findings to include detection of low level expression of the HSV-1 gD protein in TG of latently-infected mice [14]. gD is known as the primary viral protein that engages the HVEM receptor during initial infection [15]. Indeed, we have shown that HSV-1 latency and reactivation are significantly reduced in HVEM^-/-^ mice suggesting that HVEM is involved in latency-reactivation in ocularly infected mice [14]. To investigate how the absence of gD binding to HVEM affects latency-reactivation, we used a recombinant virus that does not bind to HVEM, called KOS-Rid1 [32], which was also confirmed by Connolly *et al*. [16]. KOS-Rid1 has a single mutation in aa 27 (gDQ27P) of the gD protein, which does not affect expression of gD protein [16,32].

**Fig 1:**
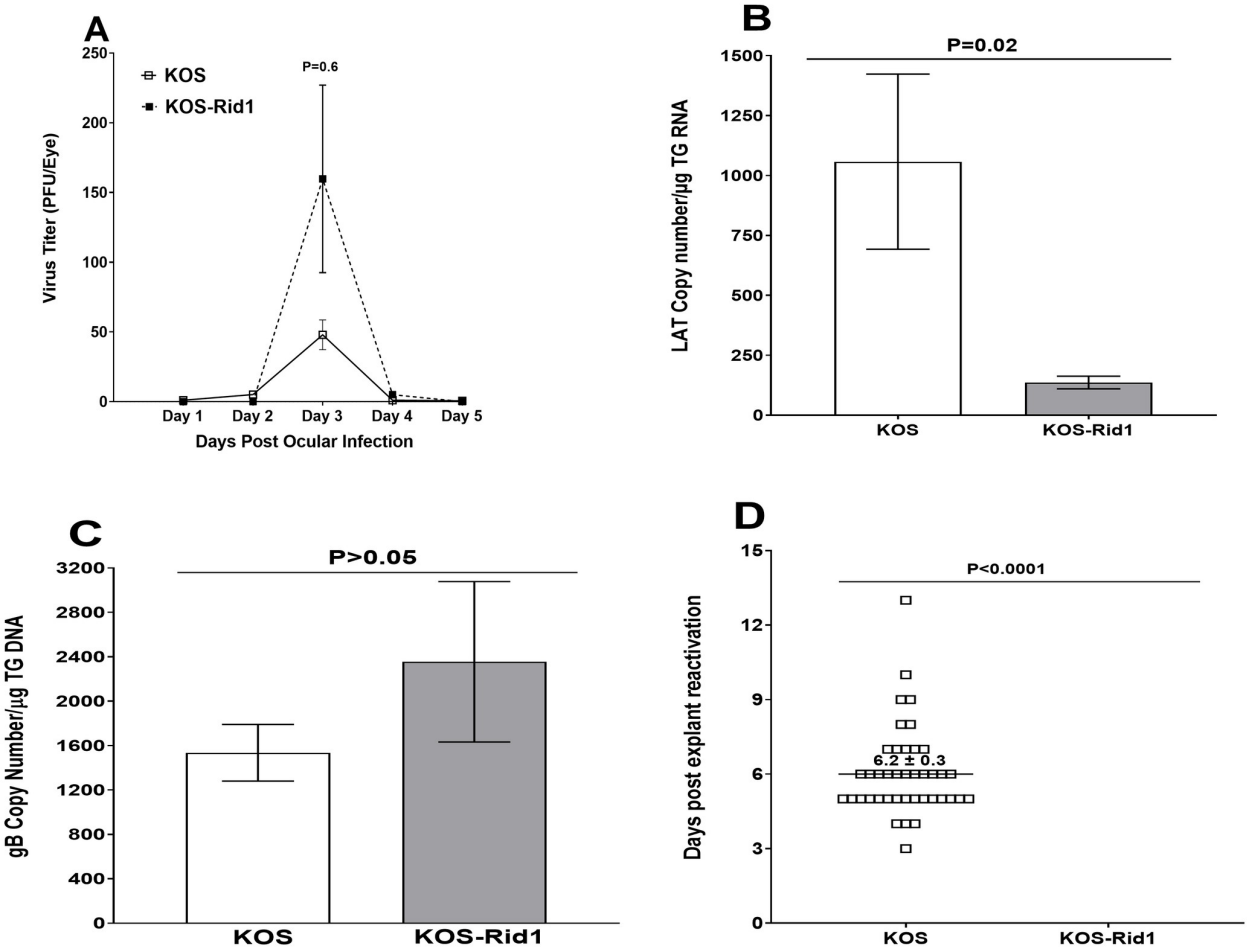
Virus titers and latency-reactivation in KOS-Rid1 infected mice. A) Virus titers in the eyes of infected mice. Twenty WT C57BL/6 mice were infected ocularly with KOS-Rid1 or KOS virus and the amount of infectious HSV-1 in tear films was determined daily by standard plaque assays as described in Materials and Methods. For each time point, the virus titer (Y-axis) represents the average of the titers from 20 eyes ± SEM; B and C) LAT and gB expression in TG of latently-infected mice. On day 28 PI, mice described above were euthanized and TG were harvested from latently-infected mice. QRT-PCR or qPCR was performed on RNA or DNA from individual mice TG. In each experiment, the estimated relative copy number of gB or LAT was calculated using standard curves generated from pGem-gB1 or pGEM-LAT5317, respectively. Briefly, DNA template was serially diluted 10-fold such that 5 μl contained from 10^3^ to 10^11^ copies of gB, or LAT then subjected to TaqMan PCR with the same set of primers. By comparing the normalized threshold cycle of each sample to the threshold cycle of the standard, the copy number for each reaction was determined. GAPDH expression was used to normalize the relative expression of gB DNA and LAT RNA in the TG. Each point represents the mean ± SEM from 20 TG for each virus; and D) Effect of gD binding on kinetics of induced reactivation in explanted TG from latently-infected mice. Twenty WT C57BL/6 mice were ocularly infected with each virus as above and 28 days PI individual TG were harvested from each group. Each TG was incubated in tissue culture media and a 10 μl aliquot was removed from each culture daily and used to infect RS cell monolayers for 10 days as described in Materials and Methods. RS cells were monitored daily for the appearance of CPE for up to 5 days to determine the time of first appearance of reactivated virus from each TG. Results are plotted as the number of TG that reactivated daily. Numbers indicate the average time that the TG from each group first showed CPE ± SEM. For each group 40 TG from 20 mice were used.

Following corneal scarification, WT C57BL/6 mice were ocularly infected with 2X10^5^ pfu/eye of KOS-Rid1 or its KOS parental virus. Viral titers in tears of 20 infected mice per virus strain were determined on days 1–5 post-infection (PI) using standard plaque assay. Virus titers were similar in mice infected with KOS and KOS-Rid1 on all days tested except on day 3 PI. On day 3 PI, virus titers in the eyes of KOS-Rid1 infected mice were higher than in the eyes of KOS infected mice but these differences were not statistically significant (Fig 1A, P = 0.6). These results suggest that the absence of gD binding to HVEM (KOS-Rid1 infected mice) does not affect virus replication in the eyes of ocularly infected mice.

How the absence of gD binding to HVEM affects levels of LAT RNA and gB DNA were determined 28 days PI by qRT-PCR and qPCR, respectively. Total TG RNA isolated from ten mice per virus-strain was used to quantify LAT RNA copy number, while DNA from TG of additional ten mice was used to quantify gB DNA copy number per virus. Cellular GAPDH RNA and DNA were used as internal controls. The amount of LAT RNA expressed during latency in KOS infected mice was significantly higher than in KOS-Rid1 infected mice (Fig 1B; p = 0.02, Fisher’s exact test). We next compared the amount of gB DNA in TG of KOS and KOS-Rid1 infected mice (Fig 1C). In contrast to the differences in LAT RNA expression in TG of KOS versus KOS-Rid1 infected mice (Fig 1B), TG from mice infected with KOS and KOS-Rid1 viral strains had similar levels of gB DNA (Fig 1C; p>0.05, Fisher’s exact test). Finally, the impact of gD binding to HVEM on reactivation of KOS and KOS-Rid1 viral strains was investigated in 20 mice from two separate experiments using an *ex-vivo* explant assay as described in Materials and Methods. In KOS infected TG, all (100%) of the 40 TG collected were reactivated, while in KOS-Rid1 mice none (0%) of the 40 TG collected were reactivated and these differences were highly significant (Fig 1D; p<0.0001, Fisher’s exact test). These results suggest that the absence of gD binding to HVEM affects levels of LAT expression and reactivation but not levels of viral DNA. Thus, in the absence of gD binding to HVEM, levels of LAT but not levels of viral DNA correlate with explant reactivation.

**Fig 2:**
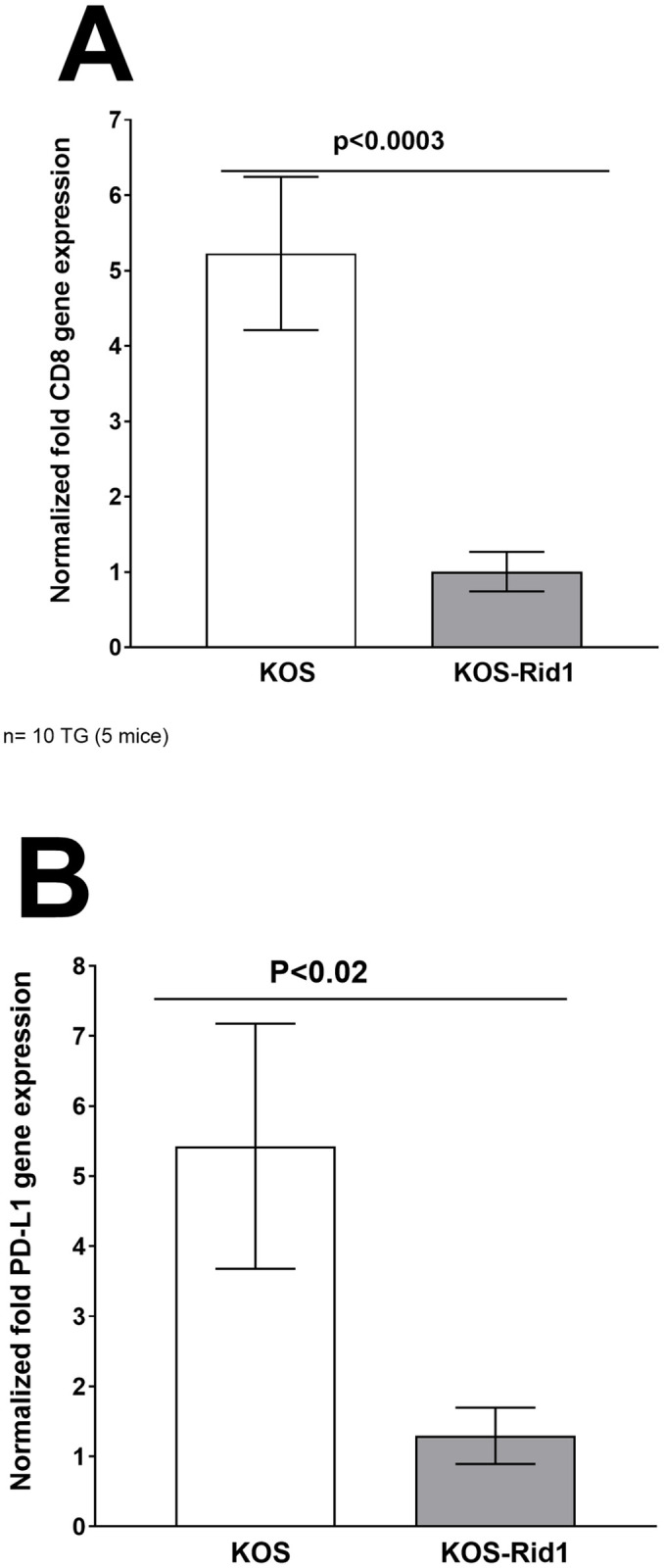
KOS-Rid1 infection affects T cell exhaustion in TG of latently-infected mice. Total TG RNA from latently-infected mice used to measure LAT expression as described in Fig 1B, were used to measure CD8 and PD-L1 expression by qRT-PCR. CD8 and PD-L1 expression in naive mice were used as a control to estimate relative expression of each transcript in TG of latently-infected mice. GAPDH expression was used to normalize the relative expression of each transcript. Each point represents the mean ± SEM from 20 TG. Panels: A) CD8 transcript; and B) PD-L1 transcript.

We previously reported that the presence of LAT contributes to higher reactivation and thus more CD8 T cell exhaustion as determined by higher expression of PD-L1 [37]. Therefore, we asked how the absence of gD binding to HVEM would affect CD8 T cell exhaustion and PD-L1 expression. Mice were infected ocularly as above with KOS and KOS-Rid1 viruses and relative levels of CD8 and PD-L1 were determined by qRT-PCR of total TG extracts on day 28 PI. The results are shown as “fold” increase compared to baseline mRNA levels in TG from uninfected naive mice (Fig 2). In TG of mice latently-infected with KOS virus, CD8 mRNA levels were significantly higher than in KOS-Rid1 infected mice (Fig 2A; p<0.0003, Fisher’s exact test). CD8 T cell exhaustion plays a major role in chronic infection and PD-L1 is a marker of T cell exhaustion [42–44]. It was therefore important to determine PD-L1 expression levels in TG of KOS and KOS-Rid1 infected mice. Similar to CD8 mRNA expression (Fig 2A), PD-L1 mRNA expression was also significantly higher in TG of KOS infected mice than in KOS-Rid1 infected mice (Fig 2B; p<0.02, Fisher’s exact test). These results confirm and extend our finding that increased levels of LAT expression correlate with higher reactivation and higher expression of both CD8 and PD-L1 transcripts in TG of KOS infected mice. Thus, higher expression of CD8 T cell mRNA in TG of latently-infected mice does not correlate with less reactivation due to these T cells being functionally exhausted. This is consistent with our previous study [37] as well as others [45,46] and contrasts with a study suggesting that CD8 T cells are involved in maintaining latency [47].

### Effect of KOS-Rid2 virus on primary infection and latency-reactivation in ocularly infected mice

**Fig 3:**
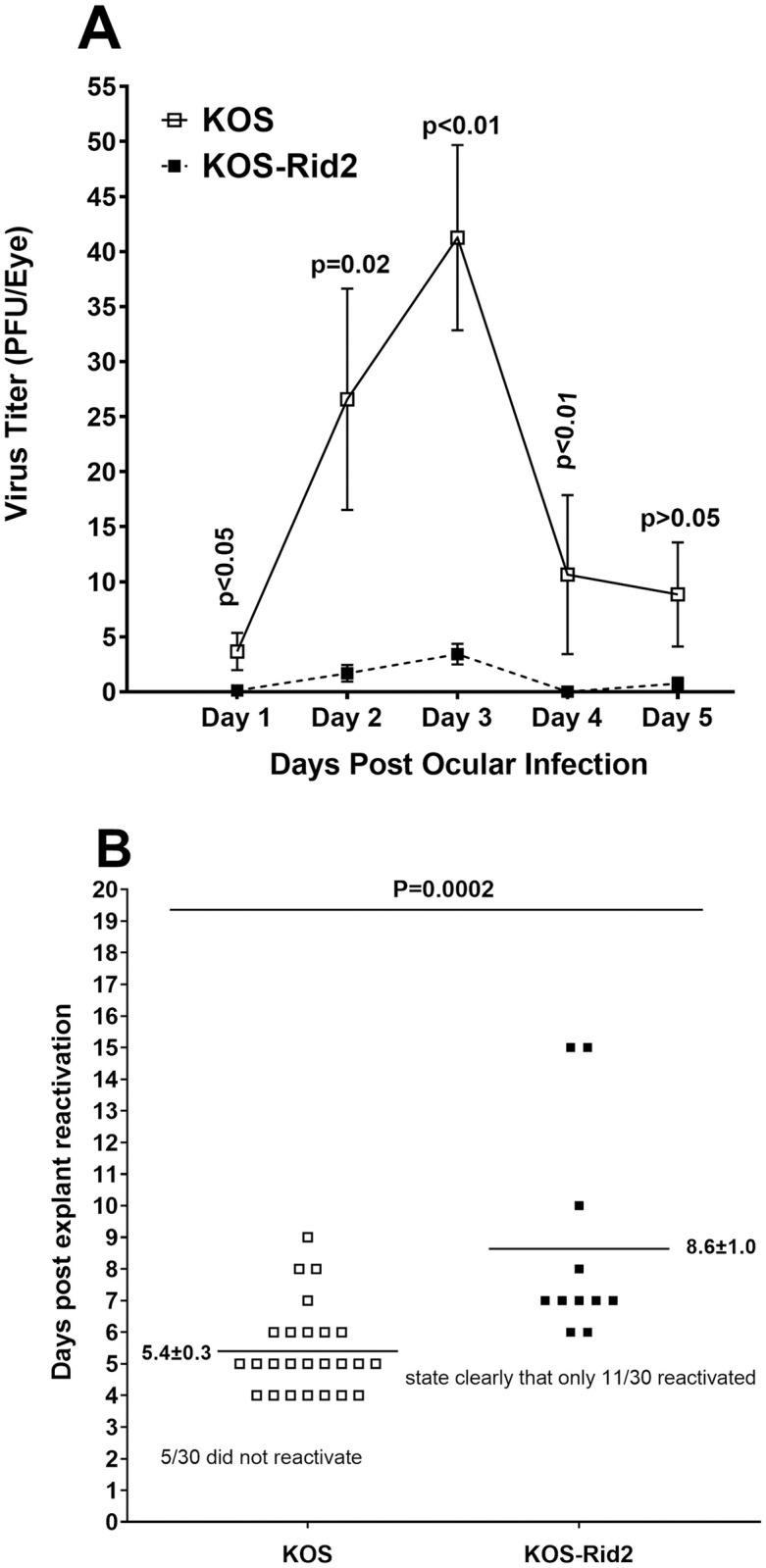
Virus titers and reactivation in KOS-Rid2 infected mice. A) Virus titers in the eyes of infected mice. WT C57BL/6 mice were infected ocularly with KOS-Rid2 or KOS control virus and the amount of infectious HSV-1 in tear films was determined daily by standard plaque assays as Fig 1. For each time point, the virus titer (Y-axis) represents the average of the titers from 20 eyes ± SEM; and B) Kinetics of induced reactivation in explanted TG from latently-infected mice. On day 28 PI, mice described above were euthanized and TG were harvested for explant reactivation as described in Fig 1D. Results are plotted as the number of TG that reactivated daily. Numbers indicate the average time that the TG from each group first showed CPE ± SEM. For each group 20 TG from ten mice were used.

The above results showed that KOS-Rid1 virus, in which glutamine at gD aa 27 was mutated to proline (Q27P), affected LAT expression, explant reactivation, and T cell exhaustion but not primary ocular infection or level of viral DNA in TG of latently-infected mice. Dean et al. [32] generated another gD mutant virus called KOS-Rid2 in which the glutamine at aa 27 of gD was mutated to arginine (Q27R) and this substitution similar to KOS-Rid1 significantly reduced virus ability to use HVEM as an entry receptor. The effect of this mutation on primary and latent infection was determined by ocular infection of 15 C57BL/6 mice with 2 X 10^5^ pfu/eye of KOS or KOS-Rid2 virus as above. Tear films were collected from 20 eyes per virus/per time point and the amount of virus titers were determined by plaque assays on rabbit skin (RS) cells (Fig 3A). KOS-Rid2 virus had a peak viral titer of approximately 5 pfu per eye with titers from days 1–4 but not day 5 being significantly lower than in KOS infected mice (Fig 3A; p>0.05, Fisher’s exact test). Virus replication in the eyes of infected mice at its peak ranges from 5 pfu/eye to 35 pfu/eye and most likely these differences are biologically significant. These results suggest that in contrast to KOS-Rid1 virus described above, gD mutation in KOS-Rid2 virus affected virus replication in the eyes of infected mice.

We next investigated whether, similar to KOS-Rid1 virus described above, the absence of gD binding to HVEM in KOS-Rid2 also correlates with reduced virus reactivation. To accomplish this, TG from the above latently-infected mice were harvested on day 28 PI and the kinetics of virus reactivation was evaluated in isolated TG. The average reactivation time for KOS infected mice was 5.3 ± 0.3 days and for KOS-Rid2 mice was 8.6 ± 1 days, and these differences were highly significant (Fig 3B, p = 0.0002, Fisher’s exact test). In the KOS infected mice 5 of 30 TG did not reactivate by day 20 post TG culture, while in KOS-Rid2 infected mice, 19 of 30 TG did not reactivate and these differences were statistically significant (p<001; Chi-squared test). Thus, in contrast to KOS-Rid1 virus that did not have any reactivation, reactivation did occur in KOS-Rid2 infected mice, but significant numbers of TG did not reactivate suggesting that these two viruses differ in their reactivation mechanisms.

### Effect of HVEM absence on explant reactivation of KOS-Rid1 and KOS-Rid2 viruses

**Fig 4:**
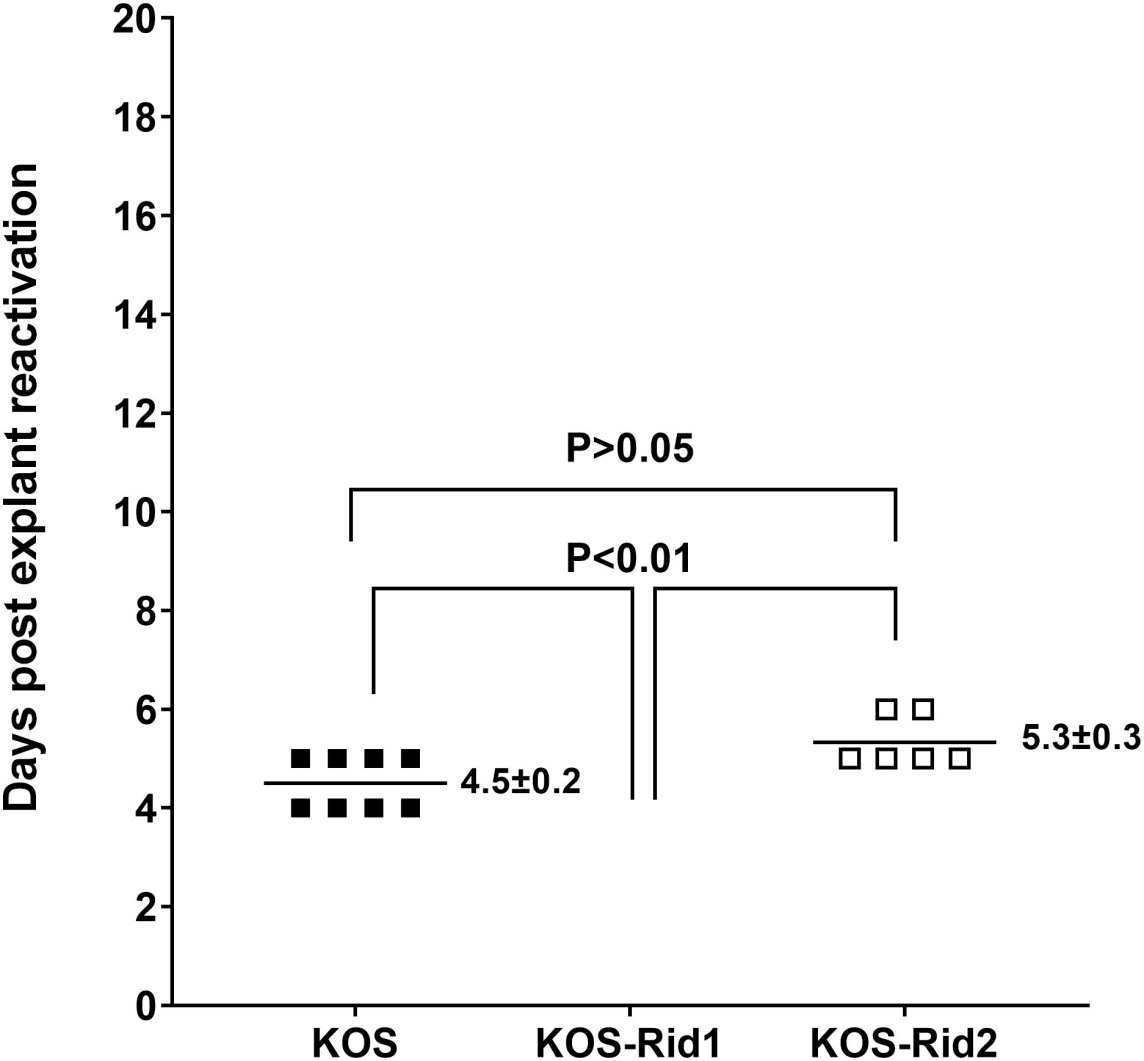
Absence of HVEM affects kinetics of induced reactivation in explanted TG from latently-infected mice. HVEM^-/-^ mice were ocularly infected with 2X10^5^ pfu/eye of KOS-Rid1, KOS-Rid2, or control KOS after corneal scarification. Twenty-eight days PI, individual TGs were isolated and incubated in tissue culture media as in Fig 1 above. Results are plotted as the number of TG that reactivated daily. Numbers indicate average time that TG from each group first showed CPE ± SEM. For each group 20 TG from ten mice were used.

The above results showed significant differences in HSV-1 reactivation in WT mice infected with KOS-Rid1 and KOS-Rid2, suggesting that the kinetics of gD binding differs in these two viruses. Thus, we investigated the impact of HVEM on explant reactivation of KOS-Rid1 and KOS-Rid2 using HVEM-deficient (HVEM^-/-^) mice as we described previously for HSV-1 strain McKrae [14]. Ten HVEM^-/-^ mice per group were ocularly infected with 2X10^5^ pfu/eye of KOS-Rid1, KOS-Rid2, or KOS virus following corneal scarification as described above. On day 28 PI, infected mice were euthanized, and their TG were used for explant reactivation. Similar reactivation was seen in TGs from mice infected with KOS and those infected with KOS-Rid2 (4.5 ± 0.2 days vs 5.3 ± 0.3 days; Fig 4; p>0.05). In contrast, no reactivation was observed in TG from KOS-Rid1 infected mice, and these differences were statistically significant (Fig 4; p<0.01; Fisher exact test). Eight of 20 TG (40%) from KOS infected mice reactivated, 6 of 20 TG (30%) from KOS-Rid2 infected mice reactivated, and 0 of 20 TG from HVEM^-/-^ mice infected with KOS-Rid1 were reactivated (Fig 4), which is similar to the reactivation seen in WT mice infected with KOS-Rid1 virus (Fig 1D, above). Differences between KOS-Rid1 infected mice compared with KOS and KOS-Rid2 infected mice were statistically significant (p<0.01). These results suggest that the absence of HVEM does not affect explant reactivation in KOS-Rid2 infected mice compared with WT KOS infected mice. However, the absence of HVEM reduces the number of reactivated TG with both viruses which is similar to our previous study using HSV-1 strain McKrae [14].

### Comparison of the levels of latency-reactivation following infection with different viruses with intact binding to gD

**Fig 5:**
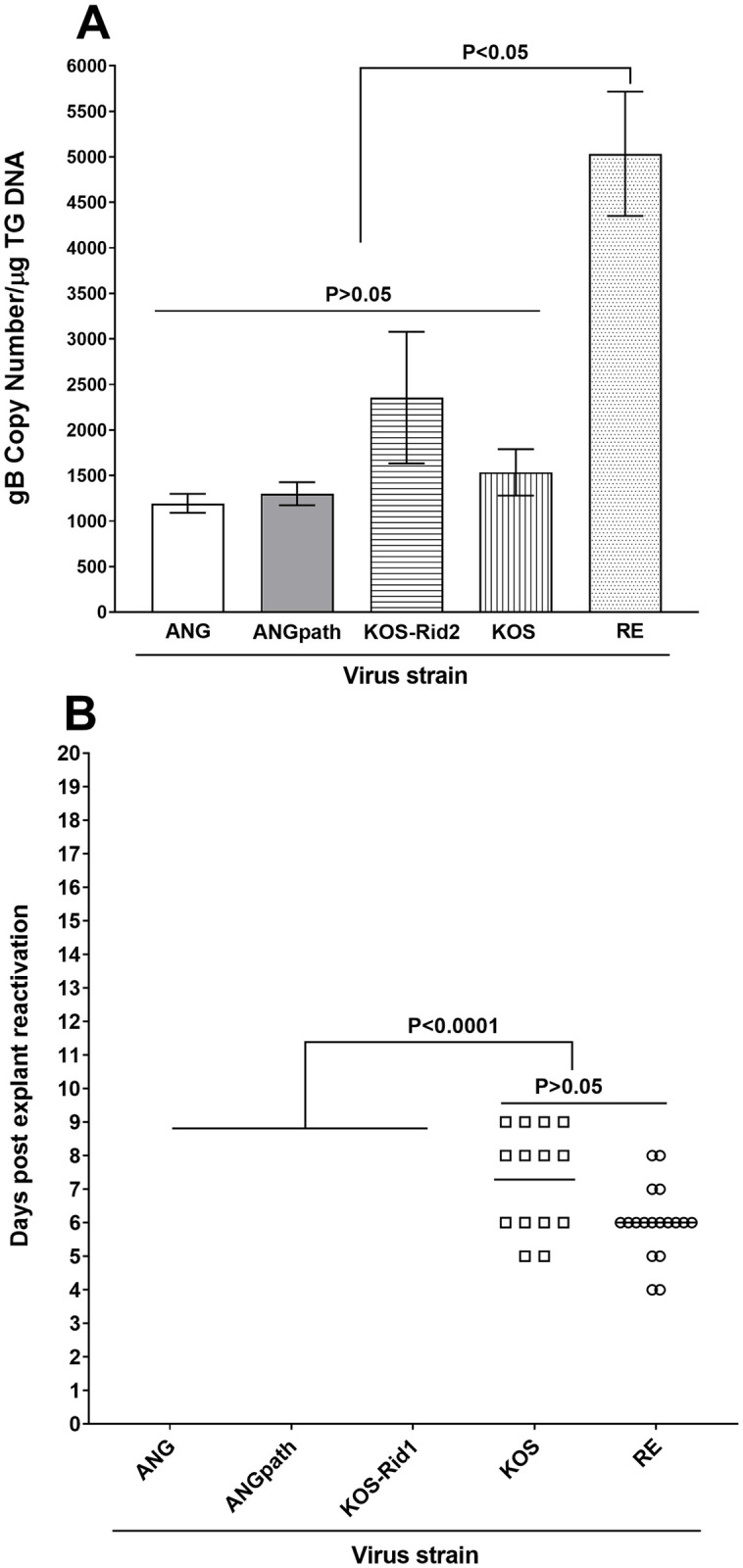
Effect of ANG and ANGpath viruses on latency-reactivation in infected mice. C57BL/6 mice were ocularly infected with HSV-1 strains ANG and ANGpath as well as KOS-Rid1, KOS-Rid2, KOS, and RE strains of HSV-1. TG from 20 mice per virus were isolated individually on day 28 PI. qPCR was performed to detect gB DNA (Panel A) and explant reactivation was used to detect reactivation (Panel B) as in Fig 1 above. TG from KOS-Rid2 infected mice was used for measuring gB DNA, while TG from KOS-Rid1 mice was used as a control for reactivation.

We next compared HSV-1 strains KOS, KOS-Rid2, and RE with the non-pathogenic HSV-1 strain ANG [48] that does not bind to gD [33], and its pathogenic mouse brain-passaged derivative ANGpath [49]. Twenty C57BL/6 mice were ocularly infected with 2X10^5^ pfu/cell of KOS, KOS-Rid2, RE, ANG, or ANGpath after corneal scarification. All infected mice survived ocular challenge as these viruses are all non-pathogenic in C57BL/6 mice. On day 28 PI, latently-infected mice were euthanized, and their TG were isolated to measure latency and reactivation. The amount of gB DNA in the TG of latently-infected mice was determined as described in Materials and Methods. Mice infected with ANG, ANGpath, KOS-Rid2, and KOS had similar amounts of gB DNA that was not significant (Fig 5A; p>0.05). In contrast, we found significantly more gB DNA in TG of RE infected mice than in TG of ANG, ANGpath, KOS-Rid2, and KOS infected mice (Fig 5A; p<0.05). These results suggest that the absence of gD binding to HVEM in KOS-Rid2, ANG, and ANGpath does not affect levels of viral DNA, which are similar to those in WT KOS infected mice.

Lastly, we asked whether, similar to KOS-Rid1 virus described above in Fig 1C, the absence of gD binding to HVEM in ANG correlates with the absence of virus reactivation. As controls we used the KOS, KOS-Rid1, ANGpath (the pathogenic form of ANG) and RE strains of HSV-1. Explant reactivation was performed on 20 TG per virus strain and reactivation was followed for 20 days post explant culture. Similar to KOS-Rid1, no reactivation (0 of 20 TG) was detected in TG of mice infected with ANG or ANGpath (Fig 5B). In KOS infected mice, 14 of 20 (70%) TG reactivated, while in RE infected mice 18 of 20 (90%) TG reactivated, and these differences were not statistically significant (Fig 5B; p>0.05). Comparison of these five strains of HSV-1 suggest that the level of viral DNA in TG does not correlate with reactivation. These results also suggest that gD from ANGpath virus may be similar to gD from ANG virus, which does not bind to HVEM.

### Absence of gD binding to HVEM reduces the levels of viral RNA but not DNA in infected RS cells

**Fig 6:**
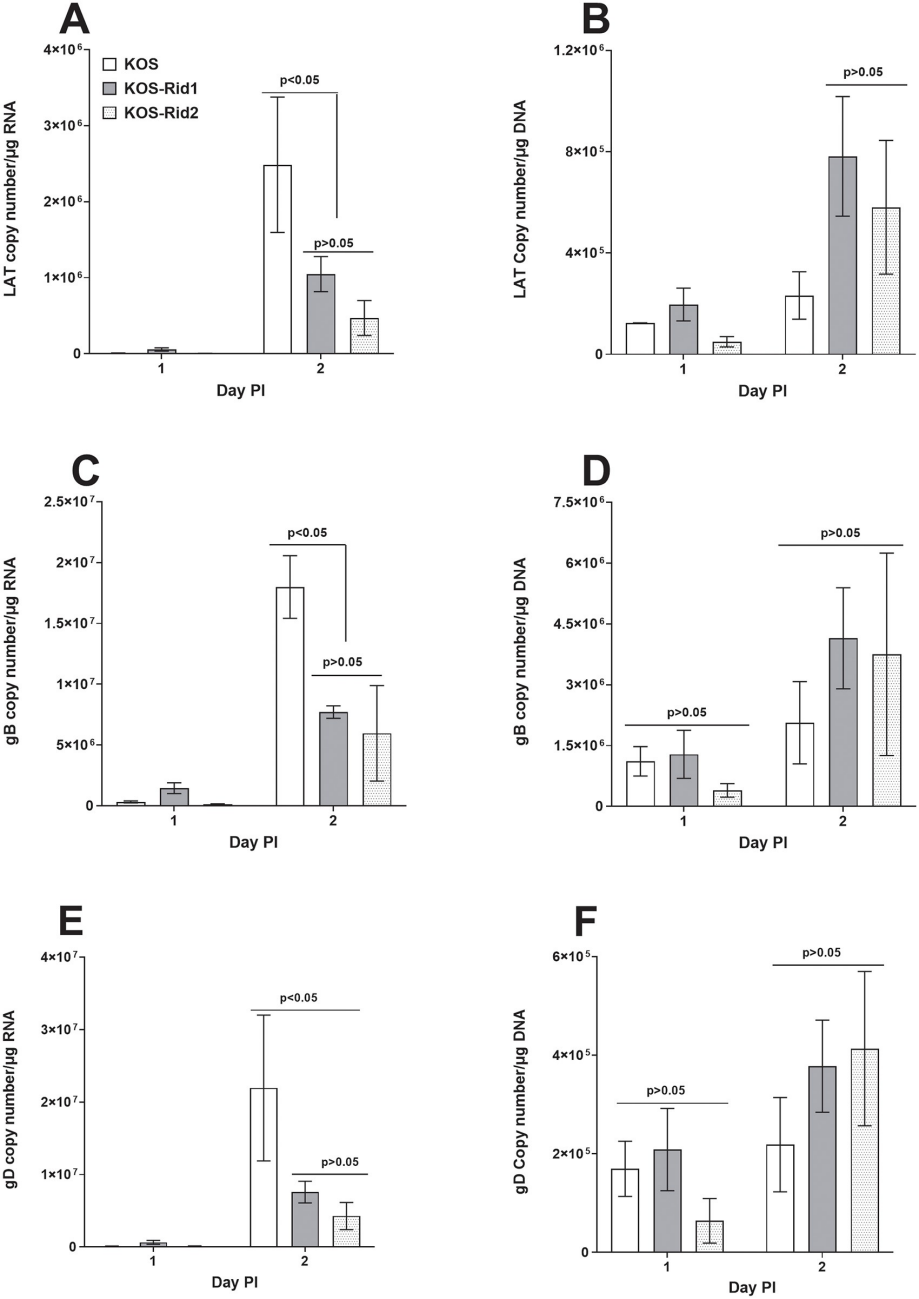
Absence of gD binding to HVEM affects levels of viral RNA but not DNA in infected RS cells. RS cells were infected with 0.04 pfu/cell of KOS, KOS-Rid1, or KOS-Rid2 viruses. Infected cells were isolated 24 and 48 hr PI and total RNA and DNA from infected cells were isolated. qPCR were performed to detect LAT, gB, and gD DNA, while qRT-PCR were performed to detect LAT, gB, and gD RNA isolated from infected cells. In each experiment, the estimated relative copy number of LAT, gB or gD was calculated using standard curves generated from pGEM-LAT5317, pGem-gB1 or, pGem-gD1, respectively as described in Fig 1 (above). Each point represents the mean ± SEM from 3 experiments.

Levels of LAT expression in TG of latently infected KOS-Rid1 mice was significantly lower than the levels of LAT in KOS infected mice (Fig 1B), while the levels of gB DNA between the two groups were not statistically significant (Fig 1C). To test if lower levels of LAT RNA compared with gB DNA in KOS-Rid1 infected mice maybe due to the absence of its binding to HVEM, we infected RS cells with KOS, KOS-Rid1 and KOS-Rid2 as described in Materials and Methods. Total RNA and DNA from infected RS cells at 24 and 48 hr PI were isolated and expression levels of gB, gD and LAT RNA and DNA were determined. Levels of viral RNA transcripts and viral DNA on 24 hr PI infection were lower compared for their levels on 48 hr PI (Fig 6, compare 24 hr versus 48 hr). Similar to our *in vivo* results (Fig 1B), levels of LAT RNA (Fig 6A), gB RNA (Fig 6C) and gD RNA (Fig 6E) for both KOS-Rid1 and KOS-Rid2 were significantly lower than for KOS infected RS cells (p<0.05; Fisher exact test). In contrast to RNA levels for LAT, gB and gD, levels of LAT DNA (Fig 6B), gB DNA (Fig 6D) and gD DNA (Fig 6F) for both KOS-Rid1 and KOS-Rid2 were similar to the level of viral DNA in KOS infected RS cells and were not statistically significant (p>0.05; Fisher exact test). Thus, both our *in vivo* and *in vitro* studies suggest that the absence of gD binding to HVEM may affect viral RNA levels and not viral DNA levels. Although our results suggest that explant reactivation is independent of the viral RNA levels, since despite the fact that both KOS-Rid1 and KOS-Rid2 have lower RNA expression levels compared with KOS virus but only KOS-Rid1 is reactivated.

## Discussion

*In vivo* studies of the mechanisms underlying HSV-1 reactivation are of paramount importance given the complex inter-relationships between replication, latency, and reactivation, as well as contributions of the microenvironment and immune responses to the status of infected sensory neurons. Multiple studies in mice have shown that viral ICP4, tk, gB, and gC lytic transcripts and their encoded proteins are expressed at very low levels in latently-infected ganglia [34–39]. We have also detected low levels of gD expression in TG of latently-infected mouse sensory neurons [14]. gD is an essential virus glycoprotein [29,50–57] encoded by the γ1 gene [58–60]. gD is highly conserved in different HSV-1 strains (*i*.*e*., KOS, F, McKrae, 17, H129, SC, ANG) [61–64], and plays a key role in virus infection and pathogenesis as it is a major inducer and target of humoral and cell-mediated immune responses following infection [65–67].

HSV-1 uses several routes of entry to initiate infection of cells including HVEM (*TNFRSF14*), nectin-1, nectin-2, 3-O-sulfated heparan sulfate (3-OS-HS), paired immunoglobulin-like type 2 receptor (PILRα), non-muscle myosin heavy chain IIA (NMHC-IIA), and myelin-associated glycoprotein (MAG) [68–72]. Previously we reported that in LAT(+) infected mice, HVEM expression was higher than in uninfected mice and reduced in LAT(-) infected mice, with no significant differences in mRNA levels of the other six known HSV receptors between LAT(+) and LAT(-) viruses [14]. In contrast to latent infection, LAT did not have a statistically significant effect on HVEM mRNA levels during the acute phase of infection [14] or low level of gD expression [23,37]. In our previous study, HSV latency and reactivation were significantly reduced in HVEM^-/-^ mice, suggesting that HVEM is involved in latency-reactivation [14]. gD is an essential viral gene [29,50–60], with a key role in virus infection and pathogenesis, and is a major inducer and target of humoral and cell-mediated immune responses [65–67]. HSV gD receptors include nectin-1 [73,74], HVEM [15–17], and 3-OS-HS [75]. We previously showed that LAT upregulates HVEM but not nectin-1, 3-OS-HS, or other HSV receptors during latency [14]. Upregulation of HVEM in TG of latently infected mice is associated with sncRNA1&2 [14,76]. Little is known about the role of HSV-1 entry receptors in latency and reactivation and what role, if any, gD may play in the process. In contrast to other known HSV-1 entry receptors [68–72], HVEM mRNA levels significantly increased in a LAT-dependent fashion in latently-infected TG of WT mice [14]. gD has been identified as the primary viral protein that engages the HVEM receptor during primary infection [15]. The virion envelope gD forms a complex with HVEM, which mimics the BTLA-HVEM interaction [77], thus allowing the virus to directly access NF-κB-dependent cell survival pathways through HVEM. In this study we looked at the role of gD binding to HVEM on latency-reactivation which, as we reported previously for HVEM upregulation in the presence of LAT [14], enhances the potential for interaction of gD with HVEM, thus promoting TG explant reactivation. *In vivo* analyses of the effect of the gD-HVEM interaction on HSV-1 latency-reactivation can be accomplished using mutated or recombinant HSV-1 viruses that lack gD binding to HVEM. Mutant viruses, including KOS-Rid1 and KOS-Rid2, as well as HSV-1 strains ANG and ANGpath all lack gD binding to HVEM and have been compared with control WT KOS to identify effects of the absence of gD binding to HVEM [16,32].

To evaluate how the HSV-1 gD interaction with HVEM affects latency-reactivation, in this study we used KOS-Rid1, KOS-Rid2, and their KOS parental virus; ANG and its pathogenic form, ANGpath; and RE viruses. Each of these strains are less virulent compared with HSV-1 strain McKrae and required corneal scarification to improve viral replication in the eyes of ocularly infected mice. We did not observe any differences in virus replication in the eyes of mice ocularly infected with KOS-Rid1, KOS-Rid2, or KOS viruses, suggesting that the aa27 mutation in KOS-Rid1 and KOS-Rid2 does not affect virus replication, which was similar to their parental KOS virus. Also, levels of viral DNA in mice ocularly infected with KOS-Rid1 and KOS-Rid2 were similar to the parental KOS virus, suggesting that the aa27 mutation does not affect viral DNA levels. In contrast to similar levels of virus replication and latency in eyes of mice infected with KOS-Rid1, KOS-Rid2, and KOS viruses, no viral reactivation was detected in KOS-Rid1 infected mice, despite increased reactivation in KOS-Rid2 and KOS infected mice. While mutation of Q27P (glutamine to proline) in KOS-Rid1 virus also affected reactivation, the Q27R (glutamine to arginine) mutation in KOS-Rid2 virus did not. These differences between mutations to proline (no charge amino acid) versus arginine (charge amino acid) is probably contributing to differences in reactivation between KOS-Rid1 with KOS-Rid2. Furthermore, the absence of reactivation in KOS-Rid1 infected mice is not due to the absence of viral gene expression, since this virus infected mice similar to its parental control KOS has been shown to grow efficiently in Chinese hamster ovary cells but not HEp-2 cells [32]. In addition, the absence of reactivation in KOS-Rid1 could be due to neurons which prematurely die after explant because LAT is not expressed. However, this is unlikely since LAT-minus viruses have reactivation but at slower rate [31,78]. These results confirm our overall hypothesis that binding of gD to HVEM is required for reactivation but does not influence viral DNA levels as determined by gB DNA levels in TG of latently-infected mice.

During latency, HSV-1 gene expression is curtailed with LAT being the only gene product that is abundantly expressed in infected mice, rabbits, and humans [1,2,79–81]. HSV-1 LAT is known to play a critical role in enhancing the reactivation phenotype [78,79,82,83] and LAT deletion mutants display significantly less reactivation in mice and rabbits [78,79,82,83]. Previously, we found that mice latently infected with wild-type HSV-1 expresses increased LAT RNA, CD8 mRNA and PD-1 mRNAs in their TG [84]. We have also shown that LAT expression in TG of mice latently infected with LAT(+), but not LAT(-) virus, contributes to increased numbers of CD8 T cells expressing PD-L1 (i.e., exhausted CD8 T cells) in the TG of these mice [37]. KOS-Rid1 and KOS viruses are both LAT(+), however, KOS-Rid1 infected TG had significantly lower levels of CD8 and PD-L1 expression than in KOS infected mice. This suggests that in the absence of gD binding to HVEM (KOS-Rid1) and in the presence of LAT, possible upregulation of HVEM does not enhance viral reactivation in TG of latently-infected mice. Similar to our previous study [14,37], lower levels of exhausted CD8 T cells correlate with lower reactivation. These results also confirm our previous study showing that the levels of latency does not correlate with time to reactivation in latently-infected TG [85]. However, this lack of correlation can depend on the virus or mouse strain. For example, the KOS and RE strains of HSV-1 both have intact gD, but the RE strain shows significantly higher latency and faster reactivation than KOS.

Similar to KOS-Rid1, ANG virus does not bind to gD [33,49]. Thus, we compared the levels of latency and reactivation in mice ocularly infected with ANG virus and its pathogenic ANGpath counterpart [49]. The amino acids sequences of HSV-1 strains 17, McKrae, KOS, ANGpath, and ANG are shown in S1 Fig. glutamine at aa27 of gD is involved in gD binding to HVEM and, similar to KOS gD, both McKrae and strain 17 viruses have a glutamine at position 27 (S1 Fig), in contrast, both ANG and ANGpath viruses have an arginine (R) at this position 27. Similar to ANG and ANGpath, in KOS-Rid2, Q at position 27 was mutated to R but this virus, in contrast to ANG and ANGpath, reactivates. Thus, R instead of Q in position 27 of gD does not correlate with the absence of reactivation in ANG and ANGpath. In addition to Q27R (ANG and ANGpath) differences with strains 17, McKrae, and KOS, three additional unique mutations (at L25P, T229I, and A345G) exist in ANG and ANGpath viruses when compared with strains 17, McKrae, and KOS. Any of these three mutations or a combination of them may contribute to blocking reactivation but not latency. Previously, it was reported that ANGpath is not pathogenic in C57BL/6 mice but is pathogenic in outbred Swiss-Webster mice using intracranial inoculation [33]. gB DNA levels in TG of ANG infected mice were similar to mice infected with ANGpath, KOS-Rid1, and KOS viruses, but all were significantly lower than gB DNA levels in the HSV-1 RE strain. Similar to KOS-Rid1, no reactivation was detected in TG of ANG and ANGpath infected mice.

Previously, the ANG gD gene was shown to encode alanine (GCC codon) at aa 84, while the ANGpath gD gene encodes glutamine (GGC codon) at this site [33]. Despite this difference, our results suggest that ANG and ANGpath have similar levels of latency and absence of reactivation. Due to mutations in the N-terminus of gD [86], ANG and ANGpath both uses nectin-1 or nectin-2 receptor for entry [86–90]. While WT HSV-1 infection is efficiently blocked by soluble forms of gD [89,90], ANG and ANGpath virus entry is not blocked by either soluble WT gD or soluble ANG gD [91]. Previously we reported that amongst the seven known HSV-1 receptors, only HVEM is upregulated in TG of latently-infected mice [14]. Thus, the absence of reactivation in TG of latently-infected ANG and ANGpath mice is consistent with our overall hypothesis that explant reactivation in the presence of LAT is associated with an interaction of gD with HVEM.

In this study, the three viruses (i.e., KOS-Rid1, ANG, and ANGpath) that do not reactivate had lower levels of latency than the three viruses that reactivated (i.e., KOS-Rid2, KOS, and RE). However, lower levels of latency can delay time to reactivate but does not completely block reactivation. Thus, the absence of reactivation could be due to the absence of gD binding to HVEM. Mice infected with these 6 viruses after corneal scarification displayed at least 20-fold less virus replication in the eyes in comparison with our previous studies using HSV-1 strain McKrae that does not need corneal scarification [14,37,76,85,92–97]. Similar fold differences were observed with regards to the levels of latency. As further proof of principal that binding of gD to HVEM is required for reactivation and this is independent of the levels of virus replication in the eyes or levels of latency in TG, we are now planning to make a similar recombinant virus as KOS-Rid1 with McKrae background that may not require corneal scarification and also may have similar virus replication in the eyes and latency in infected mice as its parental McKrae virus.

In summary, our results suggest that binding of gD to HVEM may influence reactivation but does not affect latency levels. The results of this study also establish a novel mechanism of HSV-1 reactivation in sensory neurons that could be exploited to better manage and treat latent HSV infection, and consequently virus reactivation, which is the primary cause of inflammation and subsequent eye disease.

## Materials and methods

### Ethics statement

All animal procedures were performed in strict accordance with the Association for Research in Vision and Ophthalmology Statement for the Use of Animals in Ophthalmic and Vision Research and the NIH guide for Care and Use of Laboratory Animals (ISBN 0-309-05377-3). The animal research protocol was approved by the Institutional Animal Care and Use Committee of Cedars-Sinai Medical Center (Protocols # 5030 and 8837).

### Viruses and mice

Plaque-purified HSV-1 strains, KOS, RE, KOS-Rid1, KOS-Rid2, ANG, and ANGpath viruses were grown in rabbit skin (RS) cell monolayers in minimal essential medium containing 5% fetal calf serum, as described previously [31,98]. KOS, KOS-Rid1, and KOS-Rid2 viruses were gifts from Dr. Richard Longnecker (Northwestern University). Both KOS-Rid1 and KOS-Rid2 viruses were developed in Patricia Spear’s laboratory [32]. HSV-1 strain KOS was originally isolated from Lip lesion by Kendal O Smith [99], and currently different strains of KOS are in use in different laboratories and in the current study we used the same strain of KOS as control that was used to generate KOS-Rid1 and KOS-Rid2 viruses. The HSV-1 RE strain was a gift from Dr. Robert Hendricks (University of Pittsburgh), while ANG and ANGpath were gifts from Dr. Anthony Nicola (Washington State University). The RE strain of HSV-1 originally was isolated from a human corneal lesion and has been used extensively in studies of herpetic stromal disease and in contrast to McKrae, infected animals have significantly lower incidence of mortality from encephalitis [100,101]. Overall, RE strain of HSV-1 is more pathogenic than different subtypes of KOS and both are less pathogenic than HSV-1 strain McKrae [24]. Both 6–8 weeks old male and female wild-type C57BL/6 and C57BL/6-HVEM^-/-^ mice were used in this study and were bred in-house in Cedars Sinai Medical Center. C57BL/6-HVEM^-/-^ mouse construction was described previously [14,23].

### Ocular infection

Mice were infected via the ocular route with 2 x 10^5^ pfu of each virus suspended in 2 μl of tissue culture medium (supplemented with 5% serum). Viruses were administered as an eye drop after corneal scarification.

### Titration of virus in tears of infected mice

Tear films were collected from both eyes of each ocularly infected mouse on days 1–5 post-infection (PI) using a Dacron-tipped swab [102]. Each swab was placed in 0.5 ml of tissue culture medium, squeezed, and the amount of virus was determined by a standard plaque assay on RS cells.

### *In vitro* explant reactivation assay

Mice were sacrificed at 28 days PI and individual TG were removed and cultured in tissue culture media as we described previously [103]. Media aliquots were removed from each culture daily for up to 20 days and plated on indicator RS cells to assay for the appearance of reactivated virus. As the media from explanted TG cultures were plated daily, we could determine the time at which reactivated virus first appeared in the explanted TG cultures.

### TG DNA extraction and PCR analysis for gB DNA

DNA was isolated from homogenized individual TG using the commercially available Dnaeasy Blood &Tissue Kit (Qiagen, Stanford, CA) according to the manufacturer’s instructions. PCR analyses were performed using gB specific primers (Forward—5’-AACGCGACGCACATCAAG-3’; Reverse—5’-CTGGTACGCGATCAGAAAGC-3’; and Probe—5’-FAM-CAGCCGCAGTACTACC-3’). The amplicon length for this primer set is 72 bp. Relative gB DNA copy number was calculated using standard curves generated using the plasmid pAc-gB1. In all experiments GAPDH was used to normalize transcripts.

### TG RNA extraction, cDNA synthesis, and TaqMan RT-PCR

TG from individual mice were collected on day 28 PI, immersed in RNAlater RNA stabilization reagent, and stored at -80°C until processing. Qiazol RNA reagent (Qiagen) and BCP were used to extract RNA from each individual TG. Total RNA extraction was conducted as we have described previously [104,105]. Following RNA extraction, 1000 ng of total RNA was reverse-transcribed using random hexamer primers and murine leukemia virus reverse transcriptase from the High Capacity cDNA Reverse Transcription Kit (Applied Biosystems, Foster City, CA), according to the manufacturer’s recommendations.

Primer probe sets consisted of two unlabeled PCR primers and the FAM^™^ dye-labeled TaqMan MGB probe formulated into a single mixture. The assays used in this study were as follows: 1) CD8 (α chain), ABI assay I.D. Mn01182108_m1 –Amplicon length = 67 bp; and 2) PD-L1, ABI ASSAY I.D. Mm00452054_m1 –Amplicon length = 77 bp. In all experiments glyceraldehyde-3-phosphate dehydrogenase (GAPDH, Mm99999915_g1, Amplicon size 107 bp) was used to normalize transcripts. Expression levels of transcripts in naive mice was used as a baseline control to estimate the relative expression of each transcript in TG of latently-infected mice. A custom-made primer and probe set was used for LAT as follows: Forward- 5’-GGGTGGGCTCGTGTTACAG-3’; Reverse-, 5’-GGACGGGTAAGTAACAGAGTCTCTA-3’; and Probe- 5’-FAM-ACACCAGCCCGTTCTTT-3’, Amplicon Length = 81 bp. Quantitative real-time PCR (qRT-PCR) was performed using an ABI ViiA 7 Sequence Detection System (Applied Biosystems, Foster City, CA) in 384-well plates as we described previously [104,105]. Real-time PCR was performed in triplicates for each tissue sample. The threshold cycle (Ct) values, which represent the PCR cycle at which there is a noticeable increase in reporter fluorescence above baseline, were determined using SDS 2.2 Software.

### RNA and DNA extraction from infected RS cells

RS cell monolayers at 70–80% confluency were infected with 0.04 pfu/cell of KOS, KOS-Rid1, or KOS-Rid2 viruses. Infected cells were harvested at 24 and 48 hr post infection (PI). Total RNA and DNA were isolated, and qPCR and qRT-PCR were performed using LAT and gB primers described above. The levels of gD RNA and DNA from infected RS cells were determined using custom-made gD primers and probe set as follows: forward primer, 5’-GCGGCTCGTGAAGATAAACG-3’; reverse primer, 5’-CTCGGTGCTCCAGGATAAACTG-3’; and probe, 5’-FAM-CTGGACGGAGATTACA-3’–amplicon length = 59 bp. GAPDH was used for normalization of viral RNA and DNA.

### Statistical analysis

Fisher exact test and Chi Square were performed using the computer program Instat (GraphPad, San Diego). Results were considered statistically significant when the "P" value was <0.05.

## Supporting information

S1 Fig Predicted glycoprotein D (gD) amino acid sequence of different HSV-1 strains. gD amino acid sequences for HSV-1 strains 17, McKrae, KOS, ANGPath, and ANG are shown. The putative signal sequence is underlined. Sequence differences between the five HSV-1 strains are shown in “bold” font, and the gD binding site to HVEM is indicated in bold and green font. Sequence differences between AngPath with ANG, strain 17, McKrae and KOS is indicated in yellow font.
(PDF)
