# Supplementary material for: Binding of herpesvirus entry mediator (HVEM) and HSV-1 gD affect reactivation but not latency levels
Source: PLoS Pathog. 2023 Sep 22;19(9):e1011693. doi: 10.1371/journal.ppat.1011693 (PMC10550154; doi:10.1371/journal.ppat.1011693)
Supplement: S1 Fig — gD amino acid sequences for HSV-1 strains 17, McKrae, KOS, ANGPath, and ANG are shown. The putative signal sequence is underlined. Sequence differences between the five HSV-1 strains are shown in “bold” font, and the gD binding site to HVEM is indicated in bold and green font. Sequence differences between AngPath with ANG, strain 17, McKrae and KOS is indicated in yellow font. (PDF) [file ppat.1011693.s001.pdf]

Strain17	MGGAAARLGAVILFVVIVGLHGVR**S**KYAL**V**DASLKMADPNRFRGKDLPVL
McKrae	MGGAAARLGAVILFVVIVGLHGVRGKYALADASLKMADPNRFRGKDLPVL
KOS	MGGAAARLGAVILFVVIVGLHGVRGKYALADASLKMADPNRFRGKDLPVL
ANGPath	MGGAAARLGAVILFVVIVGLHGVRGKYALADASLKMADPNRFRGKDLPV**P**
ANG	MGGAAARLGAVILFVVIVGLHGVRGKYALADASLKMADPNRFRGKDLPV**P**

Strain17	DQLTDPPGVRRVYHIQAGLPDPFQPPSLPITVYYAVLERACRSVLLNAPS
McKrae	DQLTDPPGVRRVYHIQAGLPDPFQPPSLPITVYYAVLERACRSVLLNAPS
KOS	DQLTDPPGVRRVYHIQAGLPDPFQPPSLPITVYYAVLERACRSVLLNAPS
ANGPath	D**R**LTDPPGVRRVYHIQAGLPDPFQPPSLPITVYYAVLERACRSVLLNAPS
ANG	D**R**LTDPPGVRRVYHIQAGLPDPFQPPSLPITVYYAVLERACRSVLLNAPS

Strain17	EAPQIVRGASEDVRKQPYNLTIAWFRMGGNCAIPITVMEYTECSYNKSLG
McKrae	EAPQIVRGASEDVRKQPYNLTIAWFRMGGNCAIPITVMEYTECSYNKSLG
KOS	EAPQIVRGASEDVRKQPYNLTIAWFRMGGNCAIPITVMEYTECSYNKSLG
ANGPath	EAPQIVRG**G**SEDVRKQPYNLTIAWFRMGGNCAIPITVMEYTECSYNKSLG
ANG	EAPQIVRGASEDVRKQPYNLTIAWFRMGGNCAIPITVMEYTECSYNKSLG

Strain17	ACPIRTQPRWNYYDSFSAVSEDNLGFLMHAPAFETAGTYLRLVKINDWTE
McKrae	ACPIRTQPRWNYYDSFSAVSEDNLGFLMHAPAFETAGTYLRLVKINDWTE
KOS	ACPIRTQPRWNYYDSFSAVSEDNLGFLMHAPAFETAGTYLRLVKINDWTE
ANGPath	ACPIRTQPRWNYYDSFSAVSEDNLGFLMHAPAFETAGTYLRLVKINDWTE
ANG	ACPIRTQPRWNYYDSFSAVSEDNLGFLMHAPAFETAGTYLRLVKINDWTE

Strain17	ITQFILEHRAKGSCKYALPLRIPPSACLSPQAYQQGVTVDSIGMLPRFIP
McKrae	ITQFILEHRAKGSCKYALPLRIPPSACLSPQAYQQGVTVDSIGMLPRFIP
KOS	ITQFILEHRAKGSCKYALPLRIPPSACLSPQAYQQGVTVDSIGMLPRFIP
ANGPath	ITQFILEHRAKGSCKYALPLRIPPSACLSPQAYQQGVTVDSIGMLPRFIP
ANG	ITQFILEHRAKGSCKYALPLRIPPSACLSPQAYQQGVTVDSIGMLPRFIP

Strain17	ENQRTVAVYSLKIAGWHGPKAPYTSTLLPPELSETPNATQPELAPEDPED
McKrae	ENQRTVAVYSLKIAGWHGPKAPYTSTLLPPELSETPNATQPELAPEDPED
KOS	ENQRTVAVYSLKIAGWHGPKAPYTSTLLPPELSETPNATQPELAPEDPED
ANGPath	ENQR**I**VAVYSLKIAGWHGPKAPYTSTLLPPELSETPNATQPELAPEDPED
ANG	ENQR**I**VAVYSLKIAGWHGPKAPYTSTLLPPELSETPNATQPELAPEDPED

Strain17	SALLEDPVGTVAPQIPPNWHIPSIQDAATPYHPPATPNNMGLIAGAVGGS
McKrae	SALLEDPVGTVAPQIPPNWHIPSIQDAATPYHPPATPNNMGLIAGAVGGS
KOS	SALLEDPVGTVAPQIPPNWHIPSIQDAATPYHPPATPNNMGLIAGAVGGS
ANGPath	SALLEDPVGTVAPQIPPNWHIPSIQDAATPYHPPATPNNMGLIAGAVGGS
ANG	SALLEDPVGTVAPQIPPNWHIPSIQDAATPYHPPATPNNMGLIAGAVGGS

Strain17	LLAALVICGIVYWMRR**H**TQKAPKRIRLPHIREDDQPSSHQPLFY
McKrae	LLAALVICGIVYWMRRRTQKAPKRIRLPHIREDDQPSSHQPLFY
KOS	LLAALVICGIVYWM**H**RRT**R**KAPKRIRLPHIREDDQPSSHQPLFY
ANGPath	LLAALVICGIVYWMRRRTQK**G**PKRIRLPHIREDDQPSSHQPLFY
ANG	LLAALVICGIVYWMRRRTQK**G**PKRIRLPHIREDDQPSSHQPLFY

Supplementary Fig. 1. Predicted glycoprotein D (gD) amino acid sequence of different HSV-1 strains. gD amino acid sequences for HSV-1 strains 17, McKrae, KOS, ANGPath, and ANG are shown. The putative signal sequence is underlined. Sequence differences between the five HSV-1 strains are shown in "bold" font, and the gD binding site to HVEM is indicated in bold and green font. Sequence differences between AngPath with ANG, strain 17, McKrae and KOS is indicated in yellow font.
